# The Influence of Rice Husk Ash Incorporation on the Properties of Cement-Based Materials

**DOI:** 10.3390/ma18020460

**Published:** 2025-01-20

**Authors:** Zhiyun Guo, Zhao Chen, Xurong Yang, Lanyue Zhang, Canhua Li, Chuan He, Weihong Xu

**Affiliations:** 1 School of Metallurgical Engineering, Anhui University of Technology, Maanshan 243002, China; gzy5442@163.com (Z.G.); klydl_0325@163.com (X.Y.); zly921731@163.com (L.Z.); 2 School of Materials Science and Engineering, City University of Hong Kong, Hong Kong 999077, China; zchen698-c@my.cityu.edu.hk; 3 Anhui Key Laboratory of Low Carbon Metallurgy and Solid Waste Resource Utilization, Anhui University of Technology, Maanshan 243002, China; 4 School of Mechanical and Electrical Engineering, Jiuquan Vocationl and Technical College, Jiuquan 735000, China; hchuan@outlook.com; 5 Green Environmental Protection Industry Co., Ltd., Guiyang 551109, China; wsx1983246954@163.com; 6 Guizhou Phosphorus Chemical (Group) Co., Ltd., Guiyang 551109, China

**Keywords:** rice husk ash, cement-based materials, silicon dioxide, hydration process, volcanic ash activity, mechanical properties

## Abstract

Rice husk ash is a kind of biomass material. Its main component is silicon dioxide, with a content of up to 80%. It has high pozzolanic activity and can react with hydroxide in cement. When treating rice husks, rice husk ash with high volcanic ash activity and a good microaggregate filling effect can be obtained by selecting a suitable incineration environment. These advantages make rice husk ash an ideal concrete admixture, replacing the traditional admixture such as fly ash and slag in concrete. This paper summarizes the preparation methods and physical and chemical properties of rice husk ash, as well as the physical and chemical properties of rice husk ash concrete, such as mechanical properties, temperature resistance, freezing resistance, permeability resistance and chemical erosion resistance. The results show that using 20% rice husk ash as a substitute material for cement improves the resistance strength, compressive strength, flexural strength, and permeability of concrete. In short, the incorporation of rice husk ash can effectively improve the performance of cement-based materials, which will be conducive to the green development of the building material industry and the implementation of the two-carbon strategy.

## 1. Introduction

Concrete is one of the most commonly used materials in construction. Concrete has the advantages of stability and strong reliability. In addition, its construction cost is low, the construction operation is simple, and it can meet various construction environments. So, the application of concrete is widespread [1]. But cement in concrete releases a large amount of carbon dioxide and other greenhouse gasses. This leads to a greenhouse effect and damages the climate [2,3,4].

Therefore, in order to reduce the use of cement, some mineral materials are used as supplementary cementitious materials (SCMs), for example, fly ash (FA), silica fume (SF), ground granulated blast furnace slag (GGBFS), and other minerals [5]. The mechanical properties and fracture toughness of concrete containing up to 20% FA have been improved. However, the sustainable development and long-term availability of traditional SCMs are not stable. Therefore, the concrete industry urgently needs to find alternatives to fly ash to maintain its development.

In recent years, the total output of rice in China has shown a trend in increasing year by year, and the total amount of rice husks is also increasing accordingly. The reasonable development and utilization of it has a huge role in promoting social development and economic benefits [6]. Rice husk contains a lot of silicon, so it is not easy to degrade, and it is not used as animal feed because of its low nutritional value, and its direct incineration is harmful to the environment. The resource utilization of rice husk is an important part of sustainable agricultural development, which not only helps to reduce the waste of agricultural waste, but it also can be transformed into valuable products. The ash formed under certain heating conditions can become rice husk ash (RHA) [7] with a certain use value. Rice husk ash is mainly composed of amorphous SiO_2_, which also contains carbon that has been incompletely burned and a small amount of metal oxide, meaning that rice husk ash has high volcanic ash activity and can be used as an excellent auxiliary cementing material [8,9]. Compared with mineral admixture such as fly ash, silicon ash and slag, the raw material reserve of rice husk ash is richer and its cost is lower. Therefore, the application of rice husk ash in concrete can not only help to promote the green development of the construction industry but also promote the efficient utilization of agricultural by-products, realize the resource utilization of rice husk, reduce environmental pollution, implement the concept of green development, and promote the sustainable development of the construction industry.

## 2. Preparation of Rice Husk Ash and Its Basic Properties

### 2.1. Preparation Method of Rice Husk Ash and Its Influencing Factors

The preparation methods of rice husk ash mainly include carbonization treatment, acid pretreatment, calcination, grinding, etc. The different preparation methods and process parameters have different effects on the basic properties of rice husk ash. The preparation processes of RHA are shown in Table 1.

#### 2.1.1. The Carbonization Treatment of Rice Husk Ash

Rice husk first needs to be carbonized and turned into rice husk ash, appearing as a hollow mesh of black particles. This step usually requires the control of its conditions to ensure that the rice husk is fully burned and turned to ash. Carbonized rice husk ash does not contain harmful impurities such as lead and arsenic. Rice husk ash can be obtained with a mass fraction of about 20%. It contains amorphous silica of 80–95%, and its specific surface area is about 50–60 m^2^/g [10]. The burned rice husk ash needs to be cooled, and the unburned rice husk and other large particle impurities are removed by screening to ensure the purity and quality of the rice husk ash.

#### 2.1.2. Rice Husk Ash Pretreatment

Acid pretreatment is also a very important step in the preparation of rice husk ash. Different acid pretreatment conditions can affect the characteristics of rice husk ash, so the optimal acid pretreatment conditions need to be explored in the preparation process. Before calcining the rice husk, a certain solution should be used as a pretreatment agent to soak and wash the rice husk ash so as to remove the metal oxide and some impurities [11] in rice husk ash. Acid soaking treatment can effectively remove metal oxides and some unburned residual carbon in rice husk ash, which can help improve the purity and activity of the resulting rice husk ash.

Liu et al. [12] used different pretreatment solutions; there was a large gap in the content of active silica, and the average content of active silicon dioxide treated with acid was 55% higher than that treated with alkali. The residual carbon content treated with alkali is also very high, which seriously affects the activity of rice husk ash. When the concentration of the pretreatment solution increased to 0.1 mol/L, the rice husk ash silica content also increased gradually, which was almost unchanged after 0.1 mol/L. When the pretreatment time was increased from 0.5 h to 1 h, the content of active silicon dioxide in rice husk ash increased by about 5%, and then the content of active silicon dioxide increased slowly. The most important factor affecting the appearance and chemical composition of rice husk ash was the method of rice husk pretreatment, followed by the effect of pretreatment time, then the effect of different kinds of rice husk, and finally, the effect of pretreatment concentration.

It has been concluded by Lu [11] through exploratory experiments that the best conditions for the pretreatment of rice husk ash were to ensure that the rice husk was calcined after being pretreated with acid or distilled water. The ash obtained in this way has a significantly higher percentage of soluble silicon dioxide; this method is more effective than water treatment, and the resulting silicon dioxide is more amorphous.

#### 2.1.3. Calcination Conditions and Equipment

The calcination conditions are also a key factor in determining the characteristics of rice husk ash. The best preparation conditions for highly active rice husk ash can be determined by adjusting the calcination conditions.

Wang et al. [13] prepared rice husk ash at different calcination temperatures, and the amorphous SiO_2_ in rice husk ash gradually changed to crystalline SiO_2_ from 600 °C to 800 °C with the increase in calcination temperature. It was discovered by Fapohunda’s [14] research that the various incineration processes (regarding temperature and duration) have a great impact on the content of amorphous SiO_2_, specific surface area, fineness and other properties of rice husk ash, affecting its pozzolanic activity. Rice husk ash prepared [9] by the low-temperature combustion method by Wang et al. [9] has high-purity amorphous SiO_2_, and this rice husk ash has high pozzolanic activity to treat rice husk, which can be used as an auxiliary auxiliary cementitious material in concrete. The “two-stage calcination method” is considered the most reasonable calcination system at the present stage, which plays a vital role in controlling the calcination process. This method achieves the precise control of the rice husk ash calcination process by setting two different temperature stages and strictly controlling the duration of each stage. The first stage is to forge and burn at 270–280 °C for 2 h, and the second stage is to forge and burn at 570–580 °C for 4 h. This method will not only affect the volcanic ash activity of rice husk ash but also have a direct impact on the microaggregate filling effect and the final application effect in concrete [15]. The rice husk was partially burned in the boiler at a temperature of 600 to 850 °C by Cordeiro et al. [16]. RHA is partially crystalline, composed of α quartz and an amorphous compound. After quantitative analysis, the amorphous silicon dioxide content in this RHA sample was about 49%.

Xu et al. [17] forged and fired rice husks at 600 °C for 2 h and then ground them for different times. The results showed that the fineness of rice husk ash increased gradually at first and then decreased gradually after 3 min. The rice husk ash with the best filling effect and pozzolanic activity can be obtained by grinding it in ball mill for 30 min. In summary, in order to obtain rice husk ash with high pozzolanic activity and a good microaggregate filling effect, it is necessary to precisely control the calcination and grinding process. This involves the appropriate calcination temperature, sufficient calcination time, and the optimal grinding time. In addition, the choice of equipment type will also affect the determination of the preparation process. In practical applications, the appropriate preparation process should be determined according to the specific type of equipment [18].

Rice husk ash is mainly produced on a small scale through fluidized beds. This equipment can better control the combustion process and temperature, thus ensuring the quality of rice husk ash. Fluidized bed technology can provide more a uniform heat transfer and faster combustion rate during combustion, which is crucial for maintaining the high purity of amorphous silicon dioxide in rice husk ash. This technology enables the continuous operation and stable combustion of rice husks. In this process, the change in combustion temperature will affect the microscopic morphology characteristics of rice shell ash particles. For example, the grain size of rice husk ash is larger when it is directly burned at a high temperature. In addition, the rice shell ash produced by combustion below 575 °C has amorphous silicon dioxide as the main component, and when the combustion temperature reaches 815 °C, there are crystals in the rice shell ash. The use of fluidized bed combustion technology can also reduce greenhouse gas emissions and can serve as an alternative to Portland cement and commercial silica, achieving both environmental and economic benefits.

Rice husk ash can also be prepared by mobile grate technology. This technique enables the more efficient heat transfer of rice husks during combustion, thus producing rice husk ash with specific characteristics, which is produced by mobile grate technology and is suitable for adsorbent use.

In addition, the suspension combustion technology can also be used to produce rice husk ash. In the suspension combustion process, the rice husk is boiled or suspended in the furnace, which is mainly due to the combined action of the grate vibration and the bottom-up airflow. The vibration of the grate makes the rice husk move continuously, increasing its contact area with the air; the bottom-up airflow makes the rice husk particles become suspended in the air, further increasing the mixing degree with the air. Therefore, rice husk can quickly burn and produce high-temperature flue gas, improving combustion efficiency and thermal efficiency. Maintaining a high temperature in the furnace is an important operating parameter in the combustion process of rice husks. In general, the furnace temperature is higher than the lower limit of 500 °C for the effective combustion of rice husk ash, and the combustion zone temperature is higher than 800 °C. Rice husk ash produced by this technique is often used in construction and zeolite industries. This technology allows the rice husk ash to be fully mixed with the air when burned, thus improving combustion efficiency and reducing emissions to some extent.

### 2.2. Basic Properties of Rice Husk Ash

Rice husk ash is a product obtained by burning rice husks at high temperatures [14]. Through analysis, its main mineral composition is SiO_2_. In addition, it also contains a small amount of TiO_2_, K_2_SO_4_, KCl and CaCO_3_ [19]. These components make rice husk ash have high activity and adsorption capacity, meaning it can be used in soil improvement, fertilizer additives, water treatment and other fields. The specific components are shown in Table 2. It can be seen from the table that its chemical properties are close to that of silicon ash, which can be used as an auxiliary concrete cementing material due to its high volcanic ash activity.

The arrangement of rice husk ash particles is not obviously regular, with different shapes and sizes, and most of the grains have a diameter of less than 10 μm. Particle morphology and pore structure determine the high specific surface area and high activity [27] of rice husk ash. The combustion temperature, time and its chemical composition will affect its physical properties and the volcanic ash activity. Volcanic ash has potential reactivity and can undergo secondary hydration reactions with cement hydration products, thereby further improving the microstructure of cement-based materials and enhancing their mechanical properties and durability [28] in the experimental process to achieve the best performance. Table 3 shows the basic physical properties of some rice shell ash.

**Figure 1:**
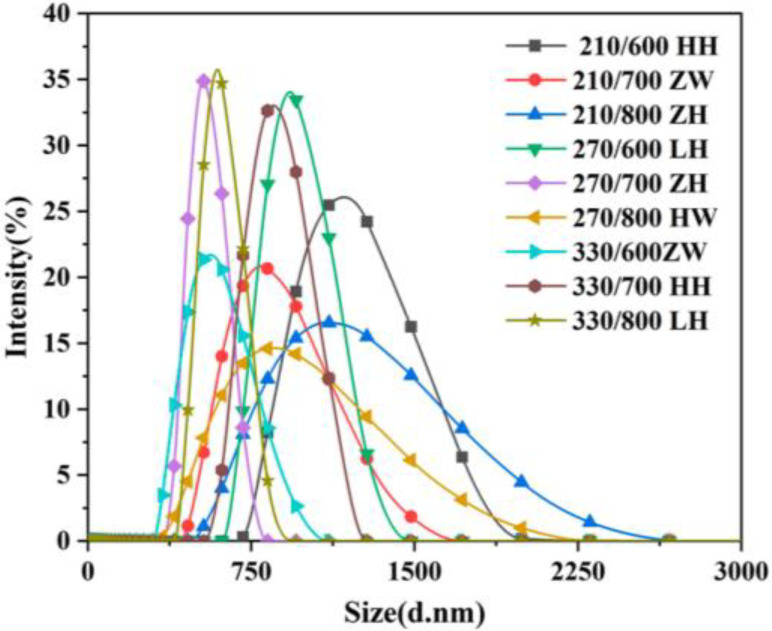
Typical particle size distribution pattern of RHA [32].

**Figure 2:**
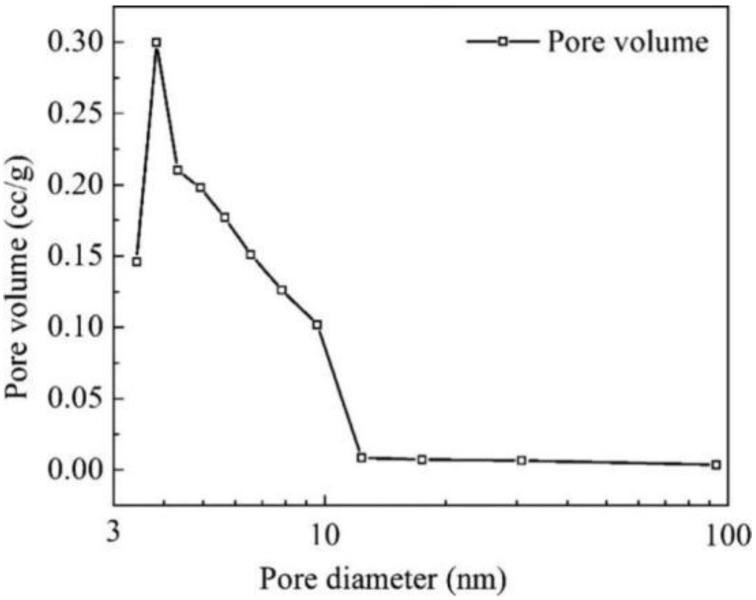
A BJH analysis of the RHA [34].

An exploration of the physical properties of RHA was made by Ma et al. [32]. Figure 1 shows typical particle size distribution patterns of RHA obtained under various processing methods. Rice husk ash has a porous microstructure [33]. As shown in Figure 2, the Barrett–Joyner–Halenda (BJH) [34] test verified the presence of nanopores of 3 nm diameter to 10 nm in RHA, explaining the high SSA and water absorption capacity of RHA, which can release absorbed water in the porous structure to improve its compressive strength.

The existence of SiO_2_ gel particles in low-temperature rice husk ash (the calcination temperature was 400 °C) was revealed, and it was found that these particles were non-tightly cohesive at the nanoscale in Ouyang et al.’s [35] research, forming a large number of voids, which not only increased the total surface area of rice husk ash but also provided more reaction sites, thus enhancing the chemical activity and adsorption capacity of rice husk ash. Moreover, RHA was estimated semi-quantitatively by the X-ray diffraction ray width method and the SiO_2_ microcrystals were found to be about ten times the size of the crystal cell, leading to the high SiO_2_ interface volume fraction in RHA and meaning it is very different to ordinary SiO_2_ crystals. This shows that the silica in rice husk ash has special structural characteristics, which can provide a basis for its unique chemical and physical properties, and can improve the compressive strength and durability of concrete.

## 3. Effect of Rice Husk Ash on Cement-Based Materials

Adding rice husk ash to concrete will affect the formation process of cement-based material and the evolution of its microstructure. The high-purity amorphous silicon dioxide in rice husk ash will react with Ca(OH)_2_ in the process of cement hydration to form C-S-H gel, which greatly improves the compactness and strength of the material and improves the overall structure of the material. With the hydration reaction, a more dense microstructure will be formed in the rice husk ash concrete. In addition, the addition of rice husk ash can partially reduce the expansion and damage level of delayed calcium (DEF), the rice husk ash concrete is more compact, and micro cracks are randomly scattered in the cement matrix and its small new structure, which improves the durability of concrete.

### 3.1. Cement Hydration Process

The cement hydration process is a series of complex chemical reactions and physical and chemical reactions, and it is a key step in the hardening, development and maturity of concrete. The hydration process directly affects the microstructure and macroscopic performance of cement-based materials, which not only determines the initial performance of concrete but also affects its long-term behavior and durability [27]. These reactions determine its strength, stability and compatibility with other materials.

The mineral composition in Portland cement clinker includes three-calcium silicate (C_3_S), dicalcium silicate (C_2_S), tricalcium aluminate (C_3_A) and tetracalcium ferric aluminate (C_4_AF). These components show different hydration characteristics after mixing with water, including the hydration rate, heat release, strength and shrinkage properties, etc. After hydration, the four mineral phases in cement are transformed into various crystalline and amorphous products, forming CH, C-S-H gel, water garnet (C_3_AH_6_) and other products [36], and hardened cement paste with a certain pore microstructure is formed. In the process of cement hydration, the initial strength of the cement depends mainly on the content of 3CaO SiO_2_, while its strength at a later stage is related to the 2CaO SiO_2_ content [17]. The content of these two compounds in Portland cement is usually between 75% and 82%. Tricalcium silicate (C_3_S) is the most important mineral component in silicate cement clinker, and its content accounts for more than 50% of the clinker. When C_3_S reacts with water, it rapidly hydrates, forming hydrated calcium silicate (C-S-H gel) and calcium dihydroxide. C-S-H gel is the main source of the strength of cement-based materials because it has excellent bonding and temperature resistance and can closely connect cement particles and aggregate to form a solid stone structure. This gel material is filled in each void of the cement hardened body, enhancing the overall strength and impermeability of the concrete. At the same time, although calcium hydroxide (CH) has a relatively small contribution to the overall strength of cement, it plays an important role in the process of cement hydration [37]. As the reaction progresses, the plasticity of cement gradually decreases, accompanied by the beginning of condensation hardening. In this process, hydration heat, a volume change and a gradual increase in strength occur. After the initial reaction, the cement hydration process continues, but it gradually slows down. The development of its strength at a later stage depends on the content of 2CaO SiO_2_. In the process of hydration, the chemical composition of cement, cement ratio, temperature and additives will affect the reaction speed.

Combining computer technology and the physical and chemical properties of cement minerals, through digital simulation, establishes the cement numerical simulation method in the process of hydration, which is used to reveal the cement’s mechanism of hydration and describe the evolution of the material structure. In addition, the further evaluation of the service performance of concrete cement base material and its structure has important significance [38].

### 3.2. Hydration Reaction Process of Rice Husk Ash Cement

Low-temperature rice husk ash has been widely studied as an auxiliary adhesive material for cement base because of its high purity of amorphous SiO_2_ and high volcanic ash activity. And it has the advantages of having large raw material reserves, a low price and amorphous SiO_2_ content up to 90%. The application of rice husk ash in cement-based materials mainly works through the micro and aggregate filling effect and volcanic ash effect. It can significantly improve the final strength of concrete, and its bonding strength and durability with a steel bar show good improvement [39,40,41]. In the process of rice husk ash, SiO_2_ can react with lime water in the process of cement hydration to generate hydrated calcium silicate with a cementing effect, which is called the volcanic ash reaction. The calcium hydroxide (CH) content in the RHA–cement cementitious system is mainly affected by the CH amount produced during the cement hydration process and the CH consumed by the volcanic ash reaction. The presence of CH has a dual influence on the performance of concrete. On the one hand, moderate CH can promote early strength development; on the other hand, excessive CH may increase the porosity inside the cement stone, affecting the durability and compressive strength of concrete. To balance this contradiction, the addition of volcanic ash material is particularly important. Low-temperature rice husk ash is a volcanic ash material containing a large amount of active silica, which can consume part of CH through the volcanic ash reaction in the process of cement hydration. This reaction not only refines the microstructure of cement stone but also enhances the later strength and durability of concrete.

When rice husk ash replaces part of the cement in the concrete, this can also cause a dilution effect and promote the hydration process of rice husk ash. Due to the huge specific surface area of rice husk ash, it has a high adsorption capacity and can absorb the free water of the mixed water, which has a certain impact on the fluidity of the concrete, but its cohesion is improved. This process of water absorption and release also plays an important role in hydration. The addition of rice husk ash will also have an impact on the compatibility of the cement concrete and the water reducing agent because the Si-O bond in the rice husk ash may coordinate with the functional group of the water reducing agent, resulting in a reduction in the active component of the water reducing agent, thus reducing the effect of the water reducing agent.

It was pointed out by Zhou et al. [42] that low-temperature rice husk ash is made of a loose adhesion of SiO_2_ particles on the nanoscale, which causes high volcanic ash activity, making it comparable to silicon ash in chemical activity, and the compressive strength of concrete is improved. The study of Mingyang et al. [43] focused on the effect of fine grinding rice husk ash on concrete, and the final results showed that rice husk ash can fully express its characteristics in concrete due to its high volcanic ash and microaggregate effects. These characteristics help to improve the chloride resistance permeability of concrete. It was found by Zheng et al. [26] that the standard consistency of the water consumption of concrete will increase with an increase in rice husk ash incorporation. Because the active ingredient in the rice husk ash reacts with the calcium hydroxide in the cement, the resulting hydration product delays the hardening process of the cement. Therefore, an appropriate amount of rice husk ash incorporation can improve the performance of concrete.

Scholars at home and abroad [44,45,46,47] have carried out a lot of research on the hydration characteristics of cement so as to establish a variety of cement hydration models, which mainly include the continuous base model and the digital image base model. These models aim to simulate and understand the hydration process of cement in different ways to predict and improve the performance of cement and its products. Upon studying the properties of rice husk ash, it was put forward by Feng [44] that the reaction kinetics of the rice husk ash and lime mixed system were consistent with the diffusion control, so the hydration process can use the Jander diffusion equation by fitting the experimental data and Jander equation, thus obtaining the reaction rate constant and diffusion coefficient and other important parameters so as to provide theoretical support for the application of rice husk ash. The hydration model was used by Nguyen [46] to describe and predict the chemical reactions and microstructural changes in cement-based materials during the hydration process. At a water–cement ratio of 0.4, Nguyen simulated the hydration process of the RHA–cement cementation system, and the microstructural changes and the evolution of calcium hydroxide content over time were observed. Based on the central particle hydration model, Narmluk and Nawa [45] have carried out specific research and improvements on the fly ash–cement cementitious system, which can quantitatively analyze the hydration process and can predict the hydration degree, hydration rate and amount of hydration products under different conditions, which provides a basis for concrete ratio design and performance optimization. Several factors influencing the hydration process, including mineral composition, the water–cement ratio, the particle diameter and temperature, were taken into account by Park [48] to obtain a more comprehensive and accurate model that can better reflect the hydration process of cement-based materials under actual conditions.

A hydration kinetics model of the RHA–cement cementitious system was established by [47] on the basis of the comprehensive consideration of the dilution effect, chemical effect and water absorption and release characteristics of RHA, which have a significant impact on the hydration process. The water–ash ratio, ambient temperature, RHA particle fineness and its mixing amount are taken as the main variables of the model. By adjusting these parameters, water absorption and release and other factors can be simulated under different working conditions. Finally, the results of the model calculations are compared with the experimental data to verify the validity and accuracy of the model. The results show that the established model can better reflect the actual hydration process. Through this model, we can predict the hydration process of the RHA–cement system under different conditions, which contributes to matching the design of such a system and quality control in engineering practice.

## 4. Physical Properties of Rice Husk Ash Cement-Based Material

### 4.1. Mechanical Properties of Rice Husk Ash Cement-Based Materials

In order to make the rice husk ash cement base material be used in daily construction and roads, rice husk ash concrete must have certain mechanical properties.

#### 4.1.1. Resistance Strength

The rice husk ash structure contains a large amount of SiO_2_, which has ultra-high volcanic ash activity, and can react with the calcium hydroxide produced by the cement clinker after hydration. Therefore, adding an appropriate amount of rice husk ash will improve the strength of the cement base material to a certain extent and improve the pore size distribution and interface structure. Therefore, the mixing amount of rice husk ash can be controlled to improve the anti-folding strength of cement-based materials, and the effect of this is more obvious in a later stage.

It was found by Zhou et al. [49] that under different curing conditions, the folding strength of cement-based materials increased first and then decreased with the addition of rice husk incorporation. It was discovered by Habeeb et al. [15] that when replacing cement with 20% rice husk cement in concrete, the folding strength of concrete can be increased by about 10–30%. The final result is that the folding strength of concrete was high when the cement was replaced with equal mass rice husk ash.

#### 4.1.2. Compressive Strength

**Figure 3:**
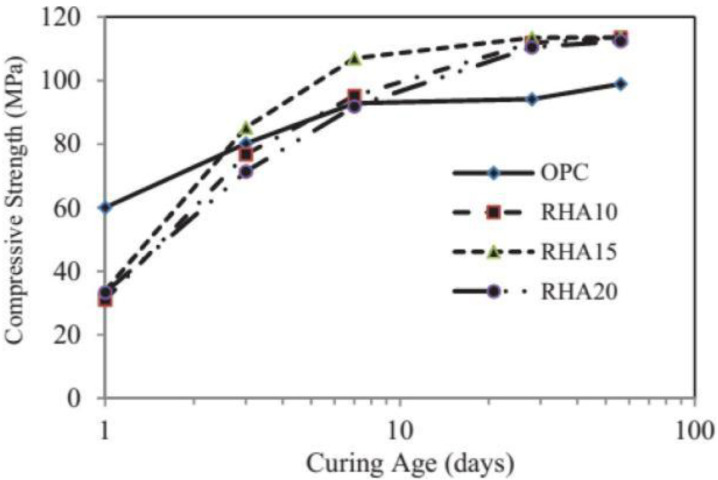
Effect of RHA on the compressive strength of the concrete [14].

It has been found by Fpohunda [14] and others that taking full advantage of the high pozzolanic activity and filling effect of rice husk ash, replacing 10–20% cement with rice husk ash, etc., can significantly improve the flexural strength of concrete, as shown in Figure 3. Different proportions of rice husk ash instead of cement incorporated into concrete were studied by Singh et al. [50], respectively, with 0%, 10%, 20%, 30% and 40% rice husk ash and other qualities of substituted cement mixed with sand to form concrete, and the final result was that the flexural strength of concrete increased first and then decreased. The use of rice husk ash instead of cement resulted in a 20% increase in compressive strength. After 28 days of curing, the compressive strength of rice husk ash concrete was 17% higher than that of ordinary concrete. Meng [51] studied the phase composition and microstructure of cement at different temperatures. The results show that the compressive strength of rice hull ash concrete is higher than that of common concrete at room temperature, and its structure is denser. The compressive strength of concrete with 20% cement replaced by rice husk ash is 50% higher than that of ordinary concrete.

It has been found in Jiang Hao’s research [52] that replacing cement with rice husk ash has a good effect on improving the compressive strength of concrete. With the improvement of the proportion of rice husk ash replacement, concrete compressive strength will improve. According to the experimental data, the best rice husk ash replacement rate was 20% and the compressive strength of concrete was at its highest at 32.0 MPa compared to the control group, increasing by 15.5%. When replacing 10% of the cement with rice husk ash, the compressive strength was 29.4 MPa, which was 6.1% higher than that of the control group. The compressive strength of the rice husk ash concrete cube was obtained by Wang et al. [53] by bringing in an empirical formula, and the compressive strength was 41.4 MPa, which was much higher than the standard compressive strength, which was due to the better compressive strength of the cement used at 28 d. Therefore, using 10–20% rice husk ash as a substitute material for cement yields the best compressive strength of concrete.

#### 4.1.3. Flexural Strength

The final tensile strength is an important standard to measure the anti-cracking strength of low-grade highway surface concrete, and it is an important factor affecting the service life of roads. Cured concrete of 7 d, 14 d and 28 d has been tested for flexural strength by general purpose testing equipment by researchers such as Singh [50]. The results show that by having 20% concrete ash instead of cement, the bending strength of concrete improves. After 28 d, the bending strength of rice husk ash concrete instead of 20% cement can be improved by 6.84%. Wang et al. [53] selected the condition of post-28 d of curing in line with the test standards of the test samples. The bending tensile strength of concrete with different amounts of rice husk ash was obtained by measuring the corresponding experimental parameters and adding them to the calculation formula of bending tensile strength. The final result showed that when rice husk ash is ground to a certain extent, continuing this grinding process produced a negative effect on the rice husk bending tensile strength of concrete ash concrete. Considering the economic and time cost, the grinding time of rice husk ash is 15 min; that is, when the average particle size of rice husk ash is 17 μm, the bending tensile strength of rice husk ash concrete is optimal. Overall, using 20% rice husk ash as a substitute material for cement improves the flexural strength of concrete.

### 4.2. Temperature Resistance

The temperature resistance of rice husk ash concrete is mainly reflected in its ability to withstand a certain degree of temperature change so that it can maintain a stable performance. This is mainly because rice husk ash is a kind of volcanic ash material, which can have a good microaggregate effect and volcanic ash effect in the concrete. The organic components in rice husk ash can absorb and release water during freezing and thawing cycles, thus improving the frost resistance of concrete. The structural damage caused by the freeze–thaw cycle can be reduced. In addition, due to the unique chemical composition and structure of rice husk ash concrete, it can improve the influence of concrete resistance to high environmental impacts to a certain extent.

Meng et al. [51] burned raw rice husk in 600 °C for 60 min to obtain rice husk ash. They used rice husk ash as raw material and carried out the RHA–concrete ratio test with different amounts. The results showed that the concrete with rice husk ash showed better compressive strength than ordinary concrete at a normal temperature and a high temperature of 800 °C. Especially at 800 °C, the concrete supplemented with 50% rice husk ash was significantly stronger than the control group without added RHA.

### 4.3. Permeability

The durability of concrete is greatly related to its impermeability. The infiltration of water is beneficial to the development of the initial concrete strength, but the chemicals inside the concrete at a later stage carry the risk of leaching, thus reducing the durability of concrete. And the hole structure is one of the important factors affecting the permeability of concrete [18]. The organic composition of rice husk ash can be well filled into the concrete structure so as to reduce the permeability of water. When rice husk ash is added to cement concrete, rice husk ash releases water continuously with a change in humidity during the formation of the cement base, thus promoting the further hydration of cement, and the SiO_2_ in the rice husk ash reacts with the calcium dihydroxide in the cement to form more hydrated calcium silicate (CSH) gels that can fill the voids in the concrete, resulting in reduced porosity [8].

It has been found by Venkatanarayanan et al. [54] that due to the honeycomb microstructure of rice husk ash particles and the wider pore size distribution of larger pore sizes, rice husk ash can quickly absorb water so as to reduce the water absorption rate of concrete, improve its impermeability, and improve its working performance. It has been discovered by Yu [55] that the structure of concrete becomes denser by changing the proportion of rice husk ash replacing cement. With an increase in rice husk ash content, the impermeability of concrete increases. And the optimal proportion of rice husk ash replacing cement is 20%.

It has been found by Liang et al. [56] that using 30% rice husk ash instead of cement decreases the water absorption of concrete, making it more compact, and it has a good protective effect on the reinforcement in buildings. It has been discovered in Saraswathy et al.’s [21] studies that adding up to a 30% replacement level of rice husk ash will reduce the permeability of concrete and improve the strength and corrosion resistance of concrete. From the above experiments, it can be seen that a certain amount of rice husk mixed instead of cement into concrete can effectively improve the seepage performance of concrete so as to improve its corrosion resistance.

## 5. Chemical Properties of Rice Husk Ash Cement-Based Material

### 5.1. Resistance to Sulfuric Acid Erosion

Sulfate erosion has a great impact on the durability of the concrete structure. Because the compound containing Ca in concrete reacts with sulfuric acid [52], the cement slurry expands, peels and softens and then deteriorates, resulting in a loss of strength and an increase in maintenance costs. If a certain amount of rice husk ash is added to the concrete, it can reduce the content of CH (calcium hydroxide) so as to reduce the production of calcium and enhance the ability of concrete to resist sulfate corrosion [8].

Through experiments on the sulfate attack on concrete with rice husk ash, it has been found by Liu et al. [57] that the addition of high pozzolanic activity could reduce the calcium dihydroxide content of concrete due to the high content of non-determinable SiO_2_ in rice husk ash. Therefore, the compactness of concrete can be improved, and the resistance to sulfuric acid corrosion of concrete can be effectively improved. It can be seen in the results of Pradhan et al.’s [58] research that concrete replaced with 10% rice husk ash has the maximum compressive strength and less strength loss after being exposed to H_2_SO_4_ for 28 d. The incorporation of rice husk ash can reduce the content of calcium hydroxide in the slurry so as to improve the impermeability of concrete, help to improve the resistance of concrete to a sulfate environment, and enhance the service life of concrete.

**Figure 4:**
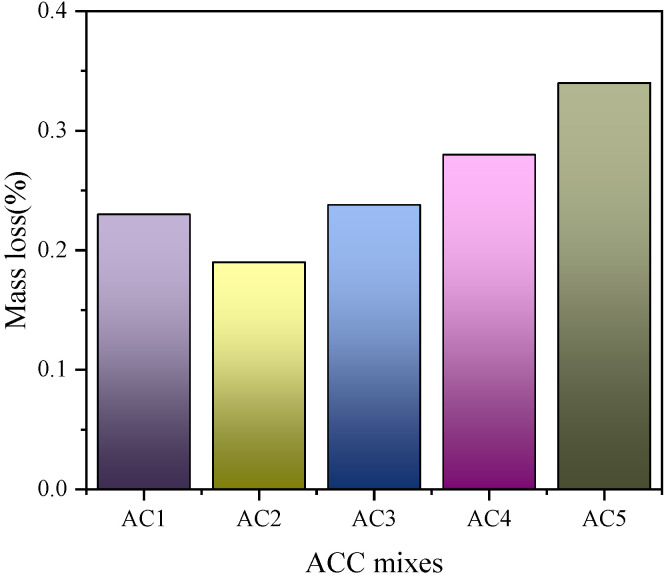
Mass loss of autoclaved lightweight aerated concrete sheet (AAC) specimens [23].

**Figure 5:**
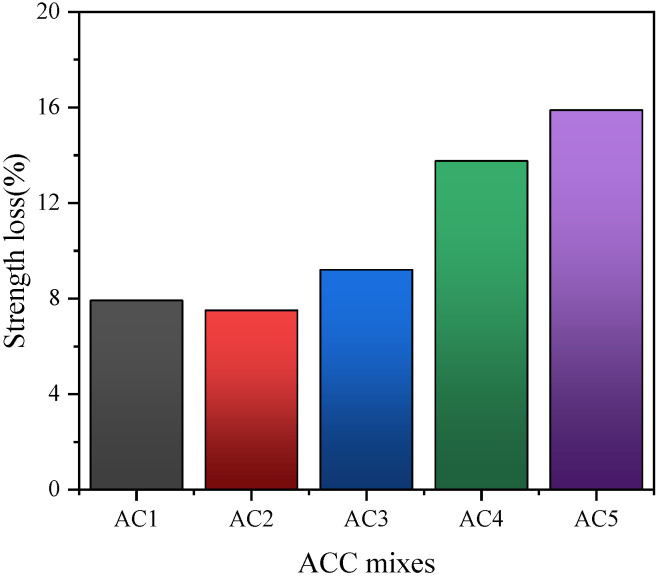
Loss of strength of autoclaved lightweight aerated concrete sheet (AAC) specimens [23].

Ground rice husk ash was mixed into cement by Chindaprasirt et al. [23] to improve the erosion of 5% sodium sulfate solution. Rice husk ash concrete is less susceptible to sulfates because of its low pH value in a sulfate environment. It was found that the sulfate corrosion resistance of 40% rice hull ash was the best. And the study found that a certain amount of ground rice husk ash can fill cracks in the concrete, increasing the density of the internal structure of the concrete. At the same time, the calcium hydroxide produced by the hydration reaction of cement can stimulate the volcanic ash effect of rice husk ash, which increases the compactness of the cementation system so as to improve the sulfate erosion resistance of concrete, as shown in Figure 4 and Figure 5. In conclusion, adding an appropriate amount of rice husk ash in concrete can improve the sulfate erosion resistance of concrete.

### 5.2. Resistance to Chloride Ion Erosion

Chloride rust corrosion erosion will cause steel corrosion. Therefore, the chloride ion diffusion performance has a great impact on the service life of concrete [23]. It has been proven in a lot of experimental studies that the appropriate amount of rice husk ash mixed in concrete can change the pore structure inside the concrete and enhance its compaction so as to improve the chloride ion permeability of the concrete.

It has been concluded by Liu et al. [59] that the properties of rice husk ash itself can improve the internal hole structure of concrete and can increase the number of adsorption and solidified chloride ion hydration products. Therefore, the incorporation of rice husk ash into concrete can improve the chloride ion permeability of concrete, and with an increase in rice husk ash incorporation, the improvement effect is better. It has been found in Mingyang et al.’s [43] electrical flux test of concrete mixed with ground rice husk ash that fine rice husk could significantly fill gaps in concrete so as to increase the compactness of the concrete structure and have a good effect on improving the chloride ion resistance of the concrete. It has been found by Wang et al. [9] that the microaggregate effect and pozzolanic effect of rice husk ash can hinder the diffusion of chloride ions and can cure and adsorb chloride ions so that it can improve the permeability performance of concrete against chloride ions. Zhang et al. [60] conducted a chloride permeability test for 28 d, and the experimental results showed that the addition of rice husk ash in concrete had a positive impact on the chloride permeability of concrete.

## 6. Conclusions

Rice husk ash concrete is a new type of building material with the advantages of environmental protection and resource reuse. Rice husk ash concrete not only has good technical performance but also performs well in terms of environmental protection and economic benefits. This paper summarizes the research background, production process, and physical and chemical properties of rice husk ash and its influence on cement-based materials.

(1) Rice husk ash contains up to 80% amorphous SiO_2_. Nanoscale SiO_2_ is loosely cohered together, meaning that rice husk ash has a huge specific surface area and high volcanic ash activity, which also makes it have a good adsorption performance.
(2) Adding 10–20% rice husk ash into cement-based materials can significantly improve the mechanical properties, temperature resistance, impermeability and chemical erosion resistance of cement-based materials and significantly improve the service life of cement-based materials.

But there are still some problems in the practical application of rice husk ash concrete.

(1) The production of rice husk ash requires high requirements for combustion equipment. However, combustion temperature control technology and by-product recovery technology are not yet perfect.
(2) Although studies have shown that rice husk ash can improve the performance of concrete, its long-term and durability still need further research and verification.
(3) At present, the research into and development of high-performance water reducing agents that match rice husk ash are still in their early stages, which limits their widespread application in concrete.
(4) Rice husk ash is mainly used in ordinary concrete but is less commonly used in other types of concrete.
(5) Although rice husk has high volcanic ash activity, its production cost and economic benefits still need further evaluation.

In the pursuit of green building and sustainable development, today, the use of rice husk ash concrete may be becoming more and more extensive, and through further research and technical improvement, it is expected that the future development of rice husk ash concrete will be more extensive and reliable.

## Figures and Tables

**Table 1:** The preparation processes of RHA.

Processing Method	Raw Material	Instrument/ Reagent	Process
Carbonization treatment	RH	—	Appearing as a hollow mesh of black particles
Acid pretreatment	RHA	Acid leaching/distilled water leaching	The percentage of soluble silica in ash content will significantly increase
Calcination conditions	Pretreated RHA	Fluidized bed/mobile grate technology/suspension combustion technology	Different calaination methods require different calcination processes
Grind	Burnt RHA	Ball mill	Gradually increase first, then gradually decrease

**Table 2:** Basic chemical properties of rice husk ash (mass fraction).

Document	Mass Fraction/%								
	SiO_2_	Al_2_O_3_	Fe_2_O_3_	CaO	MgO	SO_3_	Na_2_O	K_2_O	Loss
Cordeiro et al. [16]	82.60	0.40	0.50	0.90	—	0.10	0.10	1.80	11.90
Madandoust et al. [20]	89.61	0.04	0.22	0.91	0.42	—	0.07	1.58	5.91
Saraswathy et al. [21]	92.95	0.31	0.26	0.53	0.55	—	0.08	2.06	1.97
Sua-Iam et al. [22]	93.44	0.21	0.18	0.76	0.43	0.16	0.05	1.98	1.27
Chindaprasirt [23]	90.00	0.50	0.90	0.80	0.60	0.10	0.10	2.10	3.20
Sung-Hoon Kang [24]	92.00	0.31	0.38	0.97	0.47	—	0.20	3.87	0.76
Antiohos [25]	89.47	0.18	0.25 (ppm)	1.10	0.44	0.11	620 (ppm)	1.32	4.06
Wang et al. [13]	94.57	1.21	—	0.35	0.92	—	0.21	—	2.52
Zheng et al. [26]	83.70	1.65	1.43	3.19	—	0.93	—	—	3.19

**Table 3:** The basic physical properties of rice husk ash.

Specific Area m^2^/g	Unit Weight Kg/m³	Average Grain Diameter /μm
50–60	200.0–400.0	3.6–31.8
50–100 [29]	150.0–450.0	—
44.6 [30]	—	8.4
62.1 [31]	—	4.35

## Data Availability

Data sharing is not applicable to this article as no new data were created or analyzed in this study.
